# Functional redundancy modifies species–area relationship for freshwater phytoplankton

**DOI:** 10.1002/ece3.3512

**Published:** 2017-10-20

**Authors:** Gábor Várbíró, Judit Görgényi, Béla Tóthmérész, Judit Padisák, Éva Hajnal, Gábor Borics

**Affiliations:** ^1^ Department of Tisza Research MTA Centre for Ecological Research Debrecen Hungary; ^2^ MTA Centre for Ecological Research GINOP Sustainable Ecosystems Group Tihany Hungary; ^3^ MTA‐DE Biodiversity and Ecosystem Services Research Group Debrecen Hungary; ^4^ Department of Limnology University of Pannonia Veszprém Hungary; ^5^ MTA‐PE Limnoecology Research Group Veszprém Hungary; ^6^ Alba Regia University Center Székesfehérvár Óbuda University Székesfehérvár Hungary

**Keywords:** aquatic islands, biodiversity, island biogeography, large lake effect, macroecology, small island effect, species–area relationship

## Abstract

Although species–area relationship (SAR) is among the most extensively studied patterns in ecology, studies on aquatic and/or microbial systems are seriously underrepresented in the literature. We tested the algal SAR in lakes, pools and ponds of various sizes (10^−2^–10^8^ m^2^) and similar hydromorphological and trophic characteristics using species‐specific data and functional groups. Besides the expectation that species richness increases monotonously with area, we found a right‐skewed hump‐shaped relationship between the area and phytoplankton species richness. Functional richness however did not show such distortion. Differences between the area dependence of species and functional richness indicate that functional redundancy is responsible for the unusual hump‐backed SAR. We demonstrated that the Small Island Effect, which is a characteristic for macroscopic SARs can also be observed for the phytoplankton. Our results imply a so‐called large lake effect, which means that in case of large lakes, wind‐induced mixing acts strongly against the habitat diversity and development of phytoplankton patchiness and finally results in lower phytoplankton species richness in the pelagial. High functional redundancy of the groups that prefer small‐scale heterogeneity of the habitats is responsible for the unusual humpback relationship. The results lead us to conclude that although the mechanisms that regulate the richness of both microbial communities and communities of macroscopic organisms are similar, their importance can be different in micro‐ and macroscales.

## 1 INTRODUCTION

Space, time, energy, and matter are the most important scales at which ecosystems are organized (Lemke, 2000). The scale dependence of system attributes raised various questions in ecology. The question of how biodiversity changes over area deserved great attention among ecologists and became a widely discussed issue over the past 100 years (Arrhenius, 1921; Gleason, 1922; MacArthur & Wilson, 1967; Triantis, Guilhaumon, & Whittaker, 2012). Thousands of field evidences demonstrated that the observed or predicted number of species increases with the area surveyed. By now, it is considered almost a law in ecology and is applicable to plants, animals, and microbes (Woodcock, Curtis, Head, Lunn, & Sloan, 2006). Based on empirical observations, several models have been developed to describe the species–area relationship (SAR). Among the most widely used are the power (Arrhenius, 1921), the exponential (Gleason, 1922), the linear (Connor & McCoy, 1979), and the logistic (Archibald, 1949) models. Besides these models, several theories have also been elaborated to explain the observed shape of the SARs (Hubbell, 2001; Schmida & Wilson, 1985). Despite the huge amount of information accumulated in this field, most of our knowledge on SARs is based on analyses of macroscopic, terrestrial systems. Aquatic and especially microbial examples are rare in the literature (Bolgovics, Ács, Várbíró, Görgényi, & Borics, 2016; Fenchel & Finlay, 2005; Fierer & Jackson, 2006; Smith et al., 2005). The model framework of SAR for terrestrial systems is commonly based on the principles of island biogeography. From a biogeographical point of view, ponds and lakes are aquatic islands in a terrestrial landscape; thus, these can also be used to study island biogeographic models. In botanical studies, two orders of magnitude are usually considered as “wide range” (Dengler, 2009), while the size of the standing water bodies can vary several orders of magnitude within a relatively small geographic location. Thus, the investigation of well‐isolated aquatic islands is a promising opportunity to test biogeographic theories. Although aquatic systems provide habitats for various groups of microscopic organisms (bacterio‐, phyto‐, and zooplankton, benthic algae), these groups have received limited attention in SAR analyses so far (Bolgovics et al., 2016; Dolan, 2005) partly because of methodological difficulties (sampling, definition, and identification of species) (Rodríguez‐Ramos, Dornelas, Marañón, & Cermeño, 2014). Soininen found in a subarctic rock pool system that phytoplankton richness is related to nutrient availability, rather than to pool size (Soininen & Meier, 2014).

Planktonic algae exist as discrete individuals in the three‐dimensional water column. They form diverse assemblages that are maintained by niche partitioning, demographic stochasticity, dispersal limitation, and the physical disturbances of the aquatic environment (Borics, Várbíró, & Padisák, 2013; Chust, Irigoien, Chave, & Harris, 2013; Pueyo, 2006). Their high species richness provides an excellent model system to empirically study relevance of ecological concepts and to learn about behavior of microbial systems as phytoplankton species represent the smallest but still microscopically (mostly) identifiable and quantifiable biota.

However, estimation of species richness can be a problematic part of these kinds of investigations, because there is an extremely large size difference between the volume of sampled water body and the volume of water sample investigated. Microbial communities can never be “well‐sampled,” and thus, not surprisingly, the microbial SARs do not show asymptotic character. This implies that estimation of microbial species richness is strongly influenced by the sampling and sample processing effort (Dolan, 2005). In phytoplankton SAR studies (Smith et al., 2005; Stomp et al., 2011) in which the applied spatial scales were large enough to reveal significant trends in species richness, neither the sampling nor the abundance problem (Chao et al., 2014) could be avoided.

The aim of our study was to investigate phytoplankton SAR in a way that the area of water bodies studied covers large size scale, and the bias deriving from different richness estimations is minimized. We hypothesized that, similarly to other organisms, the area of the water bodies has a significant effect on algal species richness, and the relationship can be described by power function.

In a previous study, it was demonstrated that the abundance of algae belonging to various functional groups shows different patterns along the spatial scale (Borics et al., 2016). Therefore, we hypothesized that species richness of these groups also varies with water body size, and thus, they modify the shape of SAR curve in different ways.

## 2 MATERIALS AND METHODS

### 2.1 Site selection

The SARs can be considerably masked by differences in trophy, altitude, latitude, and other ecological characteristics of the sampled areas, which cannot be ignored during data acquisition and analyses. To avoid limitations and biases caused by heterogeneity of the water bodies and the large distance between them, we selected an area where water bodies of various sizes can be found. The Nagyiván puszta (47°27′00.36″N and 20°59′44.09″E) is a sparsely populated area in the Hortobágy National Park (Hungary), and it was used as bombing range between 1940 and 1990. Because of the intensive air‐to‐ground bombing, thousands of bomb crater ponds developed here in the range of 10^0^–10^2^ m^2^. After the bombing ended, the area was used as pasture. This led to the development of smaller (10^−2^–10^−1^ m^2^) pits and holes in the ground. The region is regularly flooded from the neighboring extended marshland (3 km^2^; Kunkápolnás), thus, due to floods and precipitation, the territory became an aquatic archipelago. The larger water bodies are perennial, while the smaller ones are temporal. However, the period when the small pits contain water is long enough for the development of algal vegetation. To increase size scale wider were also involved in the analysis of larger water bodies (oxbows, shallow reservoirs of 10^3^–10^5^ m^2^) in the Hungarian Lowland region and two large lakes (Lake Velencei and Lake Balaton). Altogether 312 samples from 64 water bodies were considered in this study.

### 2.2 Sampling

The sampling design attempted to ensure that at least five water bodies from each size categories (in the range of 10^−2^–10^2^ m^2^) are represented. The pools were selected along a north–south transect in the central part of the bombing range in each size categories. Phytoplankton samples were collected in September 2011. Surface samples were taken from the center of each crater pond by a bucket and by a small plastic dish from the small pools. In case of larger lakes, the euphotic layer was sampled. In case of these water bodies, more sampling sites within the same lake were designated and sampled, then water samples were mixed in a large container, and half a liter from the mixed water was taken for the investigations. For the analysis of phytoplankton, the samples were fixed with formaldehyde solution at a final concentration of 4% and stored in darkness at 4°C until the analyses.

Water samples taken for chemical analyses were kept in coolers (0–4°C) during transportation to the laboratory.

Samples from Lake Balaton were taken with a tube sampler; thus, samples represent the whole water column (see details in Hajnal & Padisák, 2008). As we have a low number of samples from 2011 in Lake Balaton, which could underestimate the species number due to low sampling effort, we increase the sample number (Rodríguez‐Ramos et al., 2014) with including samples from July 2003 to October 2005.

### 2.3 Morphometric variables

Four lake morphometric variables were involved in the analyses: size, volume, depth, and index of basin permanency (IBP; Kerekes, 1977). Diameters of the small pools and crater ponds were recorded by a tape measure. This variable was used to calculate the perimeter and surface area of these water bodies. Depth of the water bodies was measured with a stick ruler. In case of the larger ponds and lakes, surface area and depth data of the Hungarian National Hydrological Database were used. Using the ruler tool, the lengths of the lakes’ shoreline were measured on the Google Earth images of the lakes. Volumes of the water bodies were calculated from the lake area and mean depth data. IBP was calculated as the ratio of lake volume to shoreline length.

### 2.4 Identification and quantification of the phytoplankton taxa

Taxonomic identification of algal taxa was based on light microscopic investigation of traditional morphological features. Phytoplankton subsamples were settled (Utermöhl technique) in 1‐ or 5‐ml settling chambers for 24 hr and counted with an inverted microscope (ZEISS Axiovert‐40 CFL) at 400× magnification providing a counting error of 10% (Lund, Kipling, & Lecren, 1958). For identifying morphologically closely related taxa, the algae were investigated at higher (630× and 1,000×) magnification with traditional upright research microscope (ZEISS Axioimager A2). To detect the rare, large‐sized taxa area of the whole counting chamber was investigated at 100‐fold magnification.

### 2.5 Measurement of physical and chemical variables

Temperature, electrical conductivity, and pH were determined immediately after sampling using the appropriate electrodes (temperature and electrical conductivity: WTW LF539; pH: WTWpH 539 glass electrode). Both pH and electrical conductivity values were temperature compensated (20°C). Total phosphorus was determined by colorimetric method after digestion with H_2_SO_4_ (Chapman & Pratt, 1961).

### 2.6 Assignment to functional groups

Planktonic algae are evolutionarily adapted to the constraints of the continuously changing environment they live in, and this adaptation is reflected in their morphological and anatomical characteristics. Based on the relationship between ecological functions and algal morphology, 31 functional groups of phytoplankton were proposed by Reynolds, Huszar, Kruk, Naselli‐Flores, and Melo (2002). In this study, each species was assigned to one of these groups (see the species in appendix 1 of Borics et al., 2016).

Besides the truly planktonic organisms, the dataset contained several taxa that cannot be considered euplanktonic, for example, the benthic representatives of the diatoms and cyanobacteria groups.

### 2.7 Statistical analyses

The analyses were performed at both sample and water body level. For sample‐level analyses, actual environmental data and observed species richness values were used. For lake‐level analyses, means of the environmental variables and estimated richness values were applied. Richness estimations were based on Chao's sample‐based extrapolation curves (Chao et al., 2014). This approach is based on seamless rarefaction and extrapolation (R/E) sampling curves of three diversity metrics (richness, Shannon, and Simpson indices). To standardize the effort, estimated richness values belonging to identical sample coverage values (sc80 = 80%) were applied (Chao & Jost, 2012).

In case of lakes where the number of samples was smaller than necessary to obtain the required sample coverage, short‐range extrapolation was applied. Rarefaction curves were drawn using the iNEXT package (Hsieh et al., 2016) available in R. The applied extrapolated sample sizes exceeded less than twice the number of observed samples (Colwell et al. 2012).

Principal component analysis (PCA) was used to explore the best combinations of variables in decreasing order of explained total dispersion and to assess the relationships between variables (size‐related and chemical properties of water bodies) and objects (sample‐based and lake‐based richness values) through optimal 2‐D graphical displays. Calculations were performed by CANOCO 5.0 (ter Braak & Smilauer, 2012).

As the first PCA axis was strongly correlated with log area, in the second‐step richness values (both at the level of samples and lakes) were plotted against the area of the water bodies. Because the relationships were apparently nonlinear, we fit a Lorentzian three‐parameter peak regression model to the data using the MATLAB software. To identify the potential breakpoints where obvious changes in the slopes of response curves occur, piecewise regression was used (Toms & Lesperance, 2003). Ordinary least squares method was used to construct the regression line for the range where the increasing tendency in species density was observed. This range was defined by the break points of the broken stick regression.

### 2.8 Ethics statement

The authors state that no authority permission was needed for their study, as the study did not affect any endangered or protected species and was carried out with nondestructive methods for the habitats and the environment. The landowner of the area was the Hortobágy National Park Directorate, who approved the authors to access the area and carry out the research. The study sites were located at 47°27′00.36″N and 20°59′44.09″E coordinates.

## 3 RESULTS

**Figure 1:**
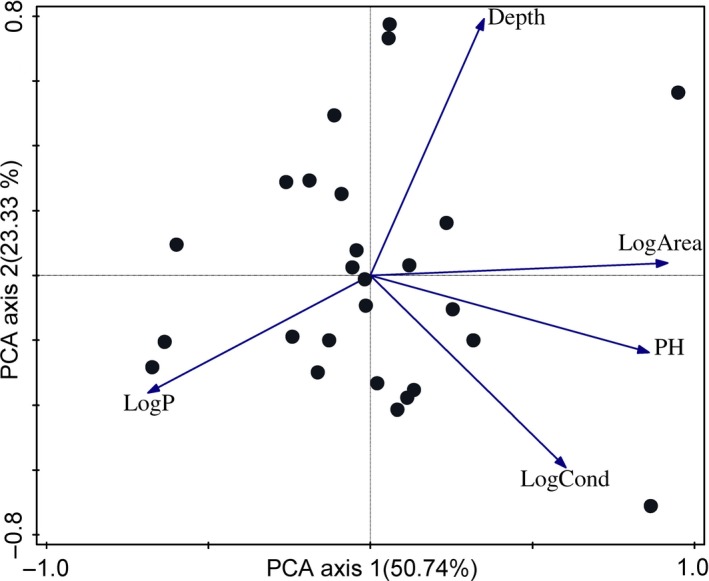
PCA diagram for the environmental variables based on all lake data. Explained cumulative variation in the first two axes: 74.07%

Results of the PCA revealed the importance of spatial variables on lakes’ phytoplankton species diversity in addition to the chemical variables (Figure 1). The first axis of the PCA accounted for 50.7% of the total variance for data and positively associated with the log area of the water bodies and negatively with phosphorus (TP) in lake‐level analysis.

**Figure 2:**
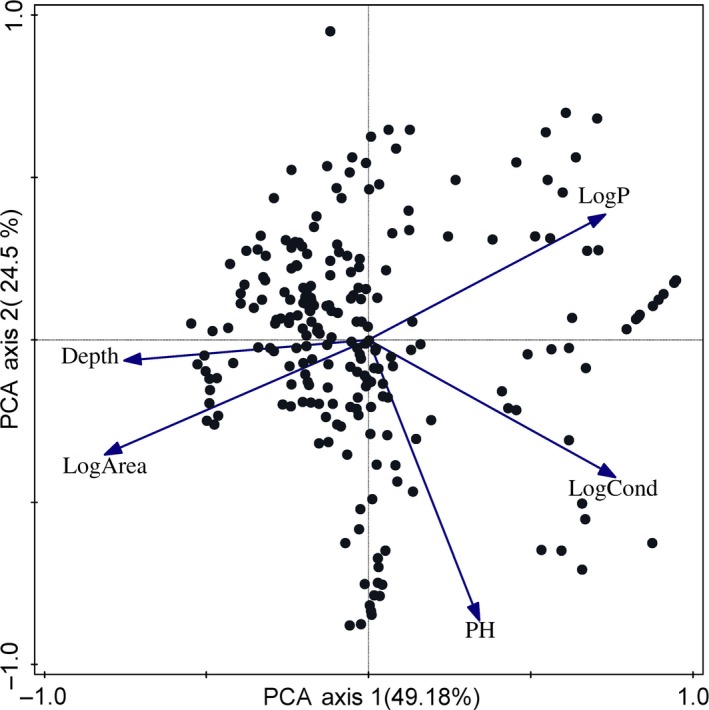
PCA diagram for the environmental variables based on sample‐level data. Explained cumulative variation in the first two axes: 73.23%

PCA analysis on sample‐level data (Figure 2) demonstrated that the F1 axis explained nearly half of the data variability (49.18%). Depth and log area had a negative loading on the first axis and had an almost completely opposite direction of TP. Eigenvalues, explained variations, and magnitudes of variable loadings of PCA are shown in Table 1.

**Table 1:** Results of the PCA analyses based on full lake dataset and on sample‐level dataset including eigenvalues, explained variations of the first four axes

	Lake based				Sample based			
	Total variation is 130.00000				Total variation is 1150.000			
Summary table								
Statistic	Axis 1	Axis 2	Axis 3	Axis 4	Axis 1	Axis 2	Axis 3	Axis 4
Eigenvalues	0.5074	0.2333	0.1306	0.088	0.4918	0.2405	0.1179	0.0867
Explained variation (cumulative)	50.74	74.07	87.12	95.92	49.18	73.23	85.03	93.69

**Figure 3:**
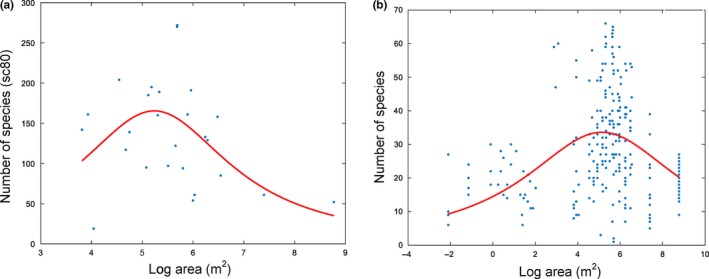
Relationship between (a) estimated and (b) observed sample‐level species richness and water body size for planktonic algae. The regression curves to the data were added using the polynomial Lorentzian peak fit. (a) a1_fit = 556.882, a2_fit = 5.23563, a3_fit = 3.36695; (b) a1_fit = 861.074, a2_fit = 5.77057, a3_fit = 27.4237

Phytoplankton species richness, both at the level of samples and lakes, showed characteristic right‐skewed hump‐shaped pattern along the spatial scale (Figure 3a,b).

In the 10^−2^–10^2^ m^2^ size range, the values showed no notable differences. In the ~10^2^ m^2^< range, they increased steadily, peaked at 10^5^–10^6^ m^2^ range, and declined thereafter.

**Figure 4:**
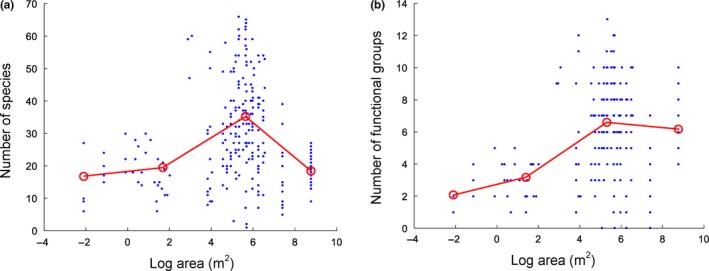
Relationship between (observed) sample‐level species richness and water body size for planktonic algae. Lines were fitted by piecewise regression, which identified the position of diversity maximum and the upper limit of the small island effect. (a) Number of estimated species at 80% sample coverage, (b) number of functional groups

These results were also corroborated by the piecewise regressions (Figure 4a).

Functional richness showed a different pattern (Figure 4b). A steady increase in the number of functional groups could be observed from the smallest water bodies up to waters of 10^5^ m^2^ without a characteristic breakpoint or critical area threshold that indicates the end of the so‐called small island effect (SIE). In contrast to species richness, where a pronounced decline of the curve was found, functional richness did not show decreasing tendency.

**Figure 5:**
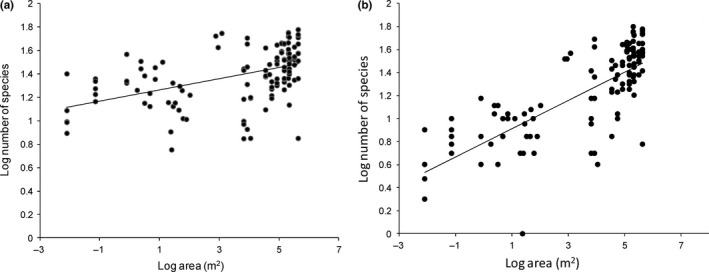
The relationship between phytoplankton species number and lake surface area in the 10^−2^–10^6^ m^2^ scale. (a) Including all species, (b) Including only true planktonic species

The SARs are characterized by the slope *z*, which describes the steepness of the relationship. This value serves as a basis for judging and comparing various SARs. Because of the observed hump‐shaped relationship, calculation of *z* value for the entire spatial scale could be misleading. Therefore, *z* was calculated for the subset of data where the increasing tendency was observed (10^−2^–10^6^ m^2^). The regression of log species number of species against log area gave slope of *z* = 0.122 (Figure 5a,b).

The question of how the various functional groups of algae contribute to the experienced humpback relationship can be answered if area dependence of the within‐group richness (i.e., the functional redundancy) is studied. Relationship between species richness of the applied functional groups and water body size showed three distinct patterns. Species richness of planktonic diatoms (**A, B, C, D**), planktonic desmids (**P**), cyanobacteria (**S1, H1, S**_**N**_) and small‐celled flagellates (X2) showed a steady increase toward the larger water bodies. Majority of the groups (**E, F, J, K, N, M, Lo, Y, X3**) had a unimodal relationship with the area showing a peak at about 10^6^ m^2^). Euglenoids (**W1** and **W2** groups) were also characterized by unimodal distribution, but their peaks occurred at smaller size range (10^4^ m^2^) (Appendix S1).

## 4 DISCUSSION

In accordance with our hypothesis that area of water bodies has a considerable effect on algal species richness, the PCA revealed a strong axis 1 related closely to water body size. Although TP also had a larger load on the first principal component, it does seem plausible that this is not a causal relationship. In a previous study, we demonstrated that nutrients had a strong inverse relationship with water body size because the smaller the water body, the larger the impact of perimeter‐related processes, such as nutrient loading from the soil to water, or the allochthonous input from the surrounding terrestrial habitats. It has recently been demonstrated that diversity has a nonlinear (humpback) relationship with phytoplankton biomass (Borics, Görgényi et al., 2014; Borics, Lukács et al., 2014; Skácelová & Lepš, 2014; Török et al., 2016), but systematic relationship between nutrients and diversity could not be established. However, we showed that surface area is a powerful variable explaining the variation in phytoplankton species richness both at sample and water body level. Although we hypothesized that species richness would steadily increase along the spatial scale, surprisingly, a pronounced hump‐shaped relationship was observed with a peak at 10^5^–10^6^ m^2^ size range. Understanding the reasons why this strange “species deficiency” occurred in the case of large lakes requires an overview of the relevant processes that influence the species richness over the lake area. Despite a huge number of papers published in this field, no coherent conclusion has yet been reached explaining the shape of the SAR curves. Reviewing the literature on this topic, Rosenzweig (1995) found that the shape of the species–area curve changes as a function of spatial scale, and on local to global scale, three distinct phases can be observed. After a sharp increase at local scale, species richness levels off at intermediate scale, and then at large‐scale, it increases again. This type of curve was shown for plants by Schmida and Wilson (1985). They argued that the shape of the curve is influenced by the combined effects of four determinants: niche relations, habitat diversity, mass effect, and ecological equivalency. The importance of these determinants changes along the scale. Niche relations that are small‐scale interactions are the most important determinants at the smallest spatial scale. Habitat diversity and the mass effect as an immigration subsidy (Hubbell, 2001) are responsible for shaping the curve at intermediate scale. On the largest spatial scale, the ecological equivalence is the most decisive factor. This latter concept refers to the fact that at the largest scales, several species with almost identical ecological properties might coexist. In other words, an increase in functional redundancy is expected. Assuming that the same actors are responsible for shaping the area dependence of phytoplankton species richness, their intensity must vary along the size scale considerably. The composition of phytoplankton mirrors the structural characteristics of the habitats. Besides species richness, functional richness also showed its peak at ~10^5^–10^6^ m^2^ water body size, which indirectly refers to the fact that system complexity has its maximum at this size range. Water bodies at 10^5^–10^6^ m^2^ range are wind‐sheltered ponds and oxbows which are spatially well‐structured aquatic environments. It has been demonstrated that under stable hydrometeorological conditions, phytoplankton shows considerable patchiness both horizontally and vertically in these water bodies reflecting to large habitat diversity within the otherwise small pelagial region of these lakes (Borics, Abonyi, Várbíró, Padisák, & T‐Krasznai, 2015; Borics et al., 2011). It has also been shown that littoral region of these waters provides various habitats even for euplanktonic species which contribute to the richness of phytoplankton (Borics et al., 2003; Krasznai et al., 2010). Thus, habitat heterogeneity as one of the most fundamental diversity‐maintenance mechanisms (Allouche & Kadmon, 2009) seems to be responsible for maintaining the highest phytoplankton richness at this size range.

While in most terrestrial ecosystems there is a positive relationship between area and habitat heterogeneity (MacArthur & Wilson, 1967), this relationship does not necessarily valid for lakes. Larger lakes have smaller littoral and pelagial ratio (Kerekes, 1977) and are subject more often to the homogenizing impact of wind stress, which results in a decrease in the number of niches. This decrease was indirectly corroborated in our investigations by the decline in the number of species of algae observed in the largest (*A* > 10^6^ m^2^) water bodies (Figure 3a,b, Appendix S1). Our results thus are in agreement with previous reports (Báldi, 2008; Myklestad & Saetersdal, 2004; Thomas et al., 2001) showing that habitat heterogeneity can override SAR. For this characteristic decline in algal species richness, we propose to use the term “large lake effect” LLE.

However, besides habitat heterogeneity, there are other reasons responsible for the higher species richness in the largest spatial scale (Connor & McCoy, 1979). Larger areas can maintain larger populations and/or metapopulations, which minimize the threat of extinction. The larger the area, the larger is the number of the random immigrants. The larger area frequently means that the system is older, where evolutionary processes are likely to occur.

As to the first reason, a critical population size below which a colonizing population has a higher risk of extinction has no relevance for algae, because having the capability of asexual reproduction, a single cell of a phytoplankton can establish a population.

Spatial dispersion is not a strong limit for microbes, even at large spatial scales (Fierer & Jackson, 2006). Planktonic algae show a range of dispersal capabilities that makes them successful and rapid colonizers (Padisák, Vasas, & Borics, 2016). They can spread by airborne transmission, water currents, animals, and by human. For the airborne dispersal, the larger destinations undisputedly have a higher chance to be inoculated, but it is not necessarily true for other vectors. Migratory birds that are considered important vectors prefer small water bodies with extended littoral region, which they can use for feeding and roosting.

The high species diversity of large ancient lakes (Lake Ochrid, the Prespa lakes) is frequently explained by the large number of endemisms. These lakes can act as evolutionary reservoirs (Albrecht & Wilke, 2008) where sympatric and/or allopatric intralacustrine speciation (Martens, Coulter, & Goddeeris, 1994; Rossiter & Kawanabe, 2000) results in evolution of new species and thus enhances diversity. Large lakes involved in this study are very young at the evolutionary time scale [Lake Balaton 15–17,000 years Korponai et al. (2011) and Tisza‐tó is a 40‐year‐old reservoir] and are not and have not been completely isolated during their history.

Results of the functional group‐level investigations revealed that richness of the algal groups strongly depends on the size of the water bodies. Not surprisingly, species with high sinking rate (**A, B, C, D, P**) showed steady increase with area. In case of the small water bodies, the fetch is too short, and the winds cannot mix the water column. Therefore, species that are not capable of active locomotion sink down from the trophic layer and disappear from the water body. Mixing increases parallel with size, and all the other groups (**H1, S1 S**_**N**_), which tolerate this physical disturbance, were represented in higher species numbers in the large lakes.

Functional groups which had a peak at their functional redundancy values at the 10^5^–10^6^ m^2^ size range can be assigned to four broad functional types. Chlorococcaleans (**F, J, X3**) and small‐celled colonial cyanobacteria (**K**) although are sensitive to sinking but much less than diatoms and can tolerate the unfavorable light conditions, which frequently occur in eutrophic lakes. The high functional redundancy of the group of flagellated algae (**E, Lo, Y**) can be accounted for by the strong light, temperature, nutrient, and other chemical gradients prevailing in these lakes (Borics et al., 2015). Flagellated algae can move across the water column and can find the optimal habitats. Most of the desmids with metaphytic origin were pooled into the group **N**. Water bodies of 10^5^–10^6^ m^2^ size range have extended littoral region with diverse submerse aquatic vegetation (Lukács et al., 2015) that provide suitable habitat for many species of desmids (Krasznai et al., 2008). These species live among the subtle leaves of macrophytes and can be easily entrained into plankton. The fourth group which showed a peak at the 10^5^–10^6^ m^2^ range was the group of *Microcystis* spp. (**M**). These taxa prefer well‐illuminated eutrophic small‐ and medium‐sized water bodies (Padisák, Crossetti, & Naselli‐Flores, 2009).

Species redundancy of **W1** and **W2** groups (euglenoids) showed a peak at 10^2^–10^4^ m^2^ range. Water bodies of this size are mostly eutrophic and macrophyte dominated. These lakes provide ideal habitats for euglenoids that prefer small‐scale spatial heterogeneity in the resources (Zakrys et al., 2004). These species frequently dominate in this kind of waters (Borics, Görgényi et al., 2014; Borics, Lukács et al., 2014) and establish diverse assemblages, therefore can be considered as typical pond‐dwellers.

Our results clearly demonstrated that species richness cannot be approximated by the well‐known power or sigmoid models because, besides the small island effect (i.e., small lake effect), another phenomenon the “large lake effect” (LLE) also influences the shape of the curve. The estimated slope (*z*) of the log species–log area curve was 0.12 for the observed data, where only the truly planktonic taxa were represented. The value for *z* is higher when isolated and lower when contiguous habitats are sampled (Bell et al., 2005). Its value calculated for communities of macroscopic organisms generally falls between 0.1 and 0.5 (Durrett & Levin, 1996), while considerably lower *z* values (0.02–0.07) were published in microbial SAR studies (Green et al., 2004; Horner‐Devine, Lage, Hughes, & Bohannan, 2004). The *z* value given in this study for phytoplankton species richness is remarkably low. Nevertheless, it is almost identical to the *z* = 0.134 value calculated by Smith et al. (2005) for phytoplankton SAR.

## 5 CONCLUSIONS

Applying identical sampling and sample processing techniques, the species–area relationship was studied for freshwater phytoplankton on a large spatial scale. Our results revealed a humpback relationship between species richness and water body size. These results imply a so‐called large lake effect (LLE), which means that in case of large lakes, wind‐induced mixing acts strongly against the habitat diversity and development of phytoplankton patchiness and finally results in lower phytoplankton species richness in the pelagial. However, functional richness did not show decline in the largest scale. Investigation of the area dependence of the functional redundancy proved that most functional groups showed the highest richness in the 10^5^–10^6^ m^2^ range, where habitat heterogeneity is the highest.

High functional redundancy of the groups that prefer small‐scale heterogeneity of the habitats is responsible for the unusual humpback relationship. The results lead us to conclude that although the mechanisms that regulate the richness of both microbial communities and communities of macroscopic organisms are similar, their importance can be different in micro‐ and macroscales.

## DATA ACCESSIBILITY

The geocoordinates of sampled ponds and lakes can be found in the Supporting information.

## CONFLICT OF INTEREST

None declared.

## AUTHORS CONTRIBUTION

GV, BG TB designed the fieldwork, and GJ, HJ made the identification. PJ, TB, VG, BG wrote and edited the MS,
