# Supplementary material for: Functional redundancy modifies species–area relationship for freshwater phytoplankton
Source: Ecol Evol. 2017 Oct 20;7(23):9905–13. doi: 10.1002/ece3.3512 (PMC5723584; doi:10.1002/ece3.3512)
Supplement: Supplementary file 1 [file ECE3-7-9905-s001.docx]

Appendix S1. Relationship between (observed) sample-level functional richness and water body size for planktonic algae. Lines are regression lines using: first column: Lorentzian peak fit, and second column: piecewise regression. Letters indicate the kodon names.

|  |  |
| --- | --- |
|  |  |
|  |  |
|   log area (m^2^) |   log area (m^2^) |
|  |  |
|  |  |
|  |  |
|   log area (m^2^) |   log area (m^2^) |
|  |  |
|  |  |
|  |  |
|   log area (m^2^) |   log area (m^2^) |
|  |  |
|  |  |
|  |  |
| ****  log area (m^2^) |   log area (m^2^) |
|  |  |
|  |  |
|  |  |
|   log area (m^2^) |   log area (m^2^) |
